# Retraction: Activation of Toll-Like Receptor-9 Promotes Cellular Migration via Up-Regulating MMP-2 Expression in Oral Squamous Cell Carcinoma

**DOI:** 10.1371/journal.pone.0288029

**Published:** 2023-06-28

**Authors:** The PLOS ONE Editors

After this article [1] was published, concerns were raised about Figures 1, 3, and 4. Specifically:

- When flipped horizontally, the 48h+CpG-ODN panel in Figure 1B in [1] appears more similar to the control panel in Fig 2a in [2] than would be expected from independent samples.
- Figure 3A in [1] is duplicated as Fig 4E in [3].
- Figure 3C in [1] is duplicated as Fig 4B in [3] and as Fig 5D in [4].
- Figure 3E in [1] is duplicated as Fig 4A in [3] and as Fig 5B in [4].
- Figure 3F in [1] appears similar to Fig 4C in [3] and to Fig 5E in [4]. In addition, when levels are adjusted to visualize background of the c-Fos panel, the bands representing the 12h and 24h results and the regions immediately surrounding these bands do not appear to match the overall background of the remainder of the panel.
- Figure 4B in [1] is duplicated as Fig 5A in [3] and as Fig 5C in [4].

In response to queries about the experiments in Figures 3A, 3C, 3E and 4F in [1], the corresponding author stated that articles [1], [3], and [4] are related to the study of the same signalling pathway (AP-1), but the downstream activators in the articles are different, and that when drafting the articles, the same representative results for the activation pathway were inadvertently selected by mistake. Article [1] was under peer review at the same time as article [3].

In response to queries about the experiments in Figure 3F, the corresponding author stated that Figure 3F in [1], Fig 4C in [3], and Fig 5E in [4] appear similar but were obtained from independent experiments and separate blots. They also noted that the irregularities in the c-Fos panels are likely the result of image or experimental artifacts. In response to queries about the 48h+CpG-ODN panel in Figure 1B in [1], the corresponding author agreed that the 48h+CpG-ODN panel in Figure 1B in [1] and the control panel in Fig 2a in [2] look similar but stated they were obtained from different experiments. The corresponding author stated that the underlying data for all results in [1] are no longer available.

In light of the above concerns with Figures 1B and 3F which question the integrity of these data, and in the absence of any underlying data required to resolve these concerns, the *PLOS ONE* Editors no longer stand by the reliability of the findings reported in [1] and therefore retract this article.

MR did not agree with the retraction. ZZ, SLi, MY, SLiu, WY, LW, and CZ either did not respond directly or could not be reached.

Owing to the concerns about similarities with previously published content [2–4], published 2010 John Wiley & Sons [2], 2012 Japanese Cancer Association [4] and 2014 Demetrios A. Spandidos Ed. & Pub. [3] which are not offered under a CC-BY license, the 48h+CpG-ODN panel in Figure 1B, Figures 3A, 3C, 3E, 3F, and Figure 4B are excluded from this article’s [1] license. At the time of retraction, the article [1] was republished to note this exclusion in the legends of Figures 1, 3 and 4 and the article’s copyright statement.
